# Rift Valley Fever Reemergence after 7 Years of Quiescence, South Africa, May 2018

**DOI:** 10.3201/eid2502.181289

**Published:** 2019-02

**Authors:** Petrus Jansen van Vuren, Joe Kgaladi, Veerle Msimang, Janusz T. Paweska

**Affiliations:** Institute for Communicable Diseases of the National Health Laboratory Service, Johannesburg, South Africa

**Keywords:** Rift Valley fever, Rift Valley fever virus, phlebovirus, arbovirus, reemerging disease, South Africa, viruses, outbreak, vector-borne infections

## Abstract

Phylogenetic analysis of Rift Valley fever virus partial genomic sequences from a patient infected in South Africa in May 2018 suggests reemergence of an endemic lineage different from that of the epidemic in South Africa during 2010–2011. Surveillance during interepidemic periods should be intensified to better predict future epidemics.

Rift Valley fever (RVF) epidemics occur at irregular intervals in Africa, on the Arabian Peninsula, in Madagascar, and on other Indian Ocean islands (*1*). South Africa has experienced 3 major epidemics during 1950–1951, 1974–1976, and 2008–2011, with smaller sporadic outbreaks in between (*2*,*3*). Phylogenetic analyses of isolates spanning 70 years have shown widespread dispersal of RVF virus (RVFV) genotypes throughout the regions where the virus is endemic and a high level of diversity within small geographic areas (*4*,*5*). We report the laboratory confirmation of 8 RVF cases in humans in South Africa in 2018 and phylogenetic analysis of the virus responsible for the outbreak.

## The Study

Communicable disease surveillance and outbreak investigation activities of the National Institute for Communicable Diseases (Johannesburg, South Africa) are approved by the Human Research Ethics Committee of the University of the Witwatersrand, Johannesburg, South Africa (M160667). In mid-May 2018, an outbreak of RVF in sheep on a farm in Free State Province, South Africa, was reported, followed by 4 probable cases in humans detected by RVFV serology (*6*). The affected farm is located in Jacobsdal District, a farming community close to the border of Northern Cape Province, where sheep are the main livestock species. In addition to the 6 patients sampled on May 21, 2018, described previously (*6*), another 4 were sampled on June 4, 2018. These patients experienced headache, muscle pain, fever, body ache, rigors, and nausea, as reported previously (*6*). A recent history of influenza-like illness was reported for only 2 of these 4 patients. All 4 lived and worked on the farm and were involved in high-risk activities, such as slaughtering, autopsying, disposal and burial of carcasses, or handling of raw meat. We obtained follow-up samples from all 10 patients for paired serologic testing (Appendix Table 1).

We performed the serologic assays, hemagglutination inhibition assay, virus neutralization test, and IgM ELISA with all serial serum samples collected from all 10 patients (*7*) and real-time reverse transcription PCR (RT-PCR) (*8*) on the serum fractions of clotted blood collected from the first 6 patients with suspected cases described previously (*6*). We extracted nucleic acid from EDTA whole blood samples collected from the initial 4 patients with probable cases using the MagMax Pathogen RNA/DNA Kit (Applied Biosystems, https://www.thermofisher.com) and then tested by RT-PCR. We determined the partial genome sequences of viruses from RT-PCR–positive whole blood samples using sequence-independent single-primer amplification combined with sequencing in triplicate on an Illumina MiSeq (https://www.illumina.com) and raw data processing, as described previously (*9*). After quality and host filtering and using a requirement of >3× coverage per base, we mapped raw reads to reference sequences representing the RVFV large (L), medium (M), and small (S) segments (GenBank accession nos. KX611605–7). We concatenated sequence fragments of segments, prepared alignments in MEGA6 (https://www.megasoftware.net), performed phylogenetic analyses using RAxML version 8.2.10 (http://evomics.org/learning/phylogenetics/raxml), and visualized trees with Figtree version 1.4.3 (http://tree.bio.ed.ac.uk/software/figtree).

Of 10 patients sampled, 8 seroconverted after 2 or 3 serial bleeds, as evidenced by a 4-fold increase in the hemagglutination inhibition assay or virus neutralization test titers (Appendix Table 1), and had RVFV-specific IgM, confirming their recent RVFV infection status. We detected RVFV RNA in EDTA whole blood samples of 3 of 4 patients sampled 7 days after estimated symptom onset (Appendix Table 1). Sequence-independent single-primer amplification sequencing yielded sequence fragments of the M and L segments in 1 (SA344-18) of 3 samples (GenBank accession nos. MH753234–41). We concatenated these partial sequence fragments (Appendix Table 2) and attained 86% (3,341/3,885 nt) of the M segment and 77.7% (4,975/6,404 nt) of the L segment. Only a single fragment was obtained of the S segment (776 nt, 45.9%) spanning nucleotides 18–793 (GenBank accession no. MH753235). Partial sequence fragments of the L segment from another sample (SA343-18) were also obtained; this sequence had a 165-bp overlap with sample SA344-18 (at nucleotides 3,276–3,440) and a single-nucleotide mismatch (A3305T, 99.4% identical) but was not included in phylogenetic analyses because of its close identity to SA344-18 and small fragment size. We prepared alignments with similarly concatenated L and M or partial S sequences from GenBank (Appendix Table 3) and a separate alignment with a 490-nt portion of the M segment of SA344-18 and sequences available from GenBank from a previous study, including sequences from the 2010 RVF outbreak in South Africa (*5*).

**Figure 1:**
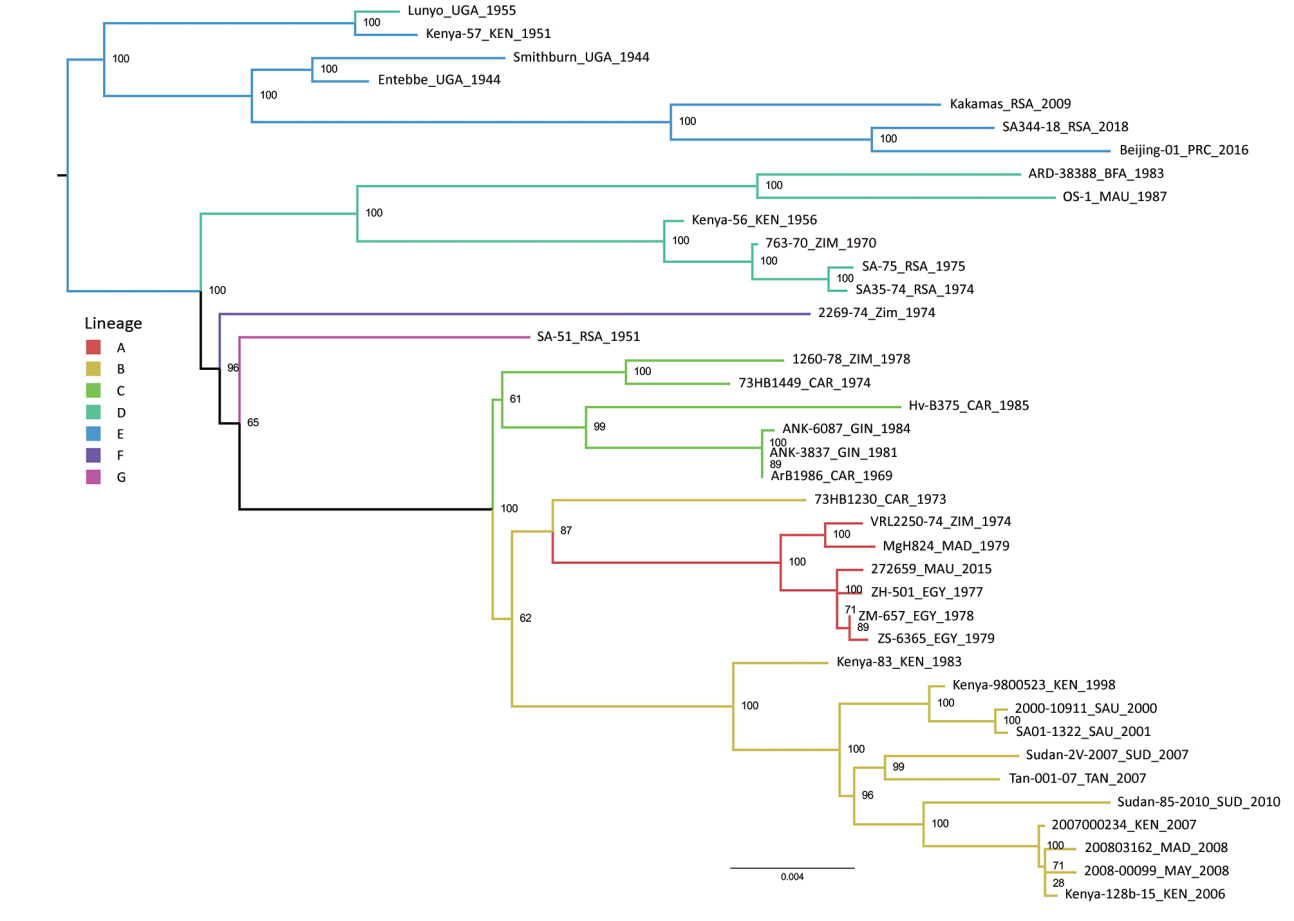
Maximum-likelihood tree showing the phylogeny of Rift Valley fever virus isolate SA344-18, collected in South Africa in May 2018, on the basis of the concatenated large segment. Lineage names according to the nomenclature of Bird et al. (*4*) are indicated. Maximum-likelihood analysis was performed in RAxML version 8.2.10 (http://evomics.org/learning/phylogenetics/raxml); 10,000 bootstrap replicates were performed. Bootstrap values are shown at nodes. Scale bar indicates nucleotide changes per base pair. BFA, Burkina Faso; CAR, Central African Republic; EGY, Egypt; GIN, Guinea; KEN, Kenya; MAD, Madagascar; MAU, Mauritania; MAY, Mayotte; PRC, China; RSA, South Africa; SAU, Saudi Arabia; SUD, Sudan; TAN, Tanzania; UGA, Uganda; ZIM, Zimbabwe.

**Figure 2:**
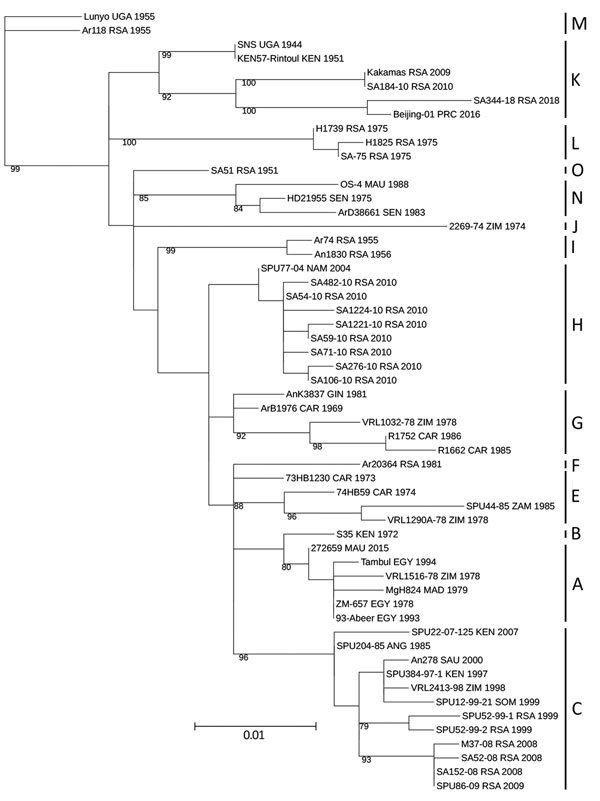
Maximum-likelihood tree showing the phylogeny of Rift Valley fever virus isolate SA344-18, collected in South Africa in May 2018, on the basis of a 490-nt fragment of the medium segment. Lineage names according to the nomenclature of Grobbelaar et al. (*5*) are indicated. Maximum-likelihood analysis was performed in RAxML version 8.2.10 (http://evomics.org/learning/phylogenetics/raxml); 100,000 bootstrap replicates were performed. Bootstrap values are shown at the nodes. Scale bar indicates nucleotide changes per base pair. ANG, Angola; CAR, Central African Republic; EGY, Egypt; GIN, Guinea; KEN, Kenya; MAD, Madagascar; MAU, Mauritania; NAM, Namibia; PRC, China; RSA, South Africa; SAU, Saudi Arabia; SEN, Senegal; SOM, Somalia; UGA, Uganda; ZAM, Zambia; ZIM, Zimbabwe.

In phylogenetic analyses of the L and M segments, isolate SA344-18 grouped with lineage E (Figure 1; Appendix Figure 1), according to the lineage nomenclature of Bird et al. (*4*). In analyses of all segments, this RVFV isolate had the closest relationship to Beijing-01, an isolate from a 2016 case exported to China from Angola (*10*), and to a 2009 sheep isolate from Kakamas, Northern Cape Province, South Africa (*11*) (Figures 1, 2; Appendix Figures 1, 2). The E lineage also included 25010-24, an isolate from a camel sampled during an outbreak in Mauritania in 2010 (Appendix Figure 1) (*11*). Nodes of the partial S segment tree were poorly supported, probably because the fragments were small (Appendix Figure 2). The overall pairwise nucleotide differences were 3.1% for L, 3.6% for M, and 3.1% for S (nonstructural protein gene), similar to values reported previously (*4*,*5*).

According to the partial 490-nt M segment tree and lineage nomenclature of Grobbelaar et al. (*5*), SA344-18 is lineage K (Figure 2). All 2010 RVFV sequences from humans in South Africa, except SA184-10, group in lineage H, distant from SA344-18. Isolate SA184-10 (lineage K) was obtained from a patient who had a needle-stick injury while vaccinating sheep with Smithburn vaccine (*5*). The 490-nt partial M sequence of SA184-10 is identical to that of the 2009 Kakamas sheep isolate (Figure 2). The only RVFV sequence from a human in Northern Cape Province (SA404-09, GenBank accession no. HM587096) grouped in lineage H (data not shown) (*5*).

## Conclusions

The major RVF epidemic in South Africa during 2010–2011 included mostly lineage H RVFV isolates, and the smaller outbreaks during the 2 preceding years included lineage C (*5*). The virus detected in Free State Province in 2018 groups in the distant lineage K, despite extensive transmission of lineage H during 2010–2011 in the same province. The 2018 isolate, SA344-18, detected in the absence of a major epidemic, is closely related to viruses from isolated cases, such as Kakamas-2009 and Beijing-01. These isolates group in the lineage that includes Smithburn vaccine viruses and its parent strain, Entebbe, isolated in Uganda in 1944 (*4*). Camel isolate 25010-24 also falls in this lineage, despite most other RVFV isolates from Mauritania grouping with distant lineages (*11*).

Intensified surveillance during interepidemic periods would enhance our knowledge of the genetic evolution of RVFV. Isolated outbreaks, similar to the one reported here, probably have occurred more frequently than recorded. Establishment of sentinel herds of susceptible animals and increased vector surveillance could yield isolates more frequently and lead to a better characterization of the strains maintained at low levels between epidemics. Our results indicate an ongoing activity and evolution of RVFV during interepizootic periods and highlight the importance of a cryptic transmission cycle that enables establishment of endemicity, which at times inevitably emerges in the form of an explosive outbreak.

Appendix Additional information on Rift Valley fever reemergence after 7 years of quiescence, South Africa, May 2018.
