# Supplementary material for: Rift Valley Fever Reemergence after 7 Years of Quiescence, South Africa, May 2018
Source: Emerg Infect Dis. 2019 Feb;25(2):338–41. doi: 10.3201/eid2502.181289 (PMC6346436; doi:10.3201/eid2502.181289)
Supplement: Appendix — Additional information on Rift Valley fever reemergence after 7 years of quiescence, South Africa, May 2018. [file 18-1289-Techapp-s1.pdf]

Article DOI: https://doi.org/10.3201/eid2502.181289

Rift Valley Fever Reemergence after 7 Years of Quiescence, South Africa, May 2018

Appendix

Appendix Table 1. Laboratory results of samples obtained from patients with suspected RVFV infections, South Africa, May 2018*

Patient no.	Sample reference no.	Date of symptom onset†	Dates of sample collection	PCR whole blood (C_t)‡	RVFV HAI test result	RVFV IgM ELISA result	RVFV VNT result
1	SA342-18	Mid-May 2018	2018 May 21	ND	Negative	Negative	Negative
	SA391-18		2018 Jun 4		Negative		Negative
2	SA343-18	Mid-May 2018	2018 May 21	Positive (34.94)	1:40	Positive	Negative
	SA392-18		2018 Jun 4		1:640		1:20
	SA429-18		2018 Jun 19		1:5,120		1:40
3	SA344-18	Mid-May 2018	2018 May 21	Positive (31.87)	1:640	Positive	1:20
	SA386-18		2018 Jun 4		1:2,560		1:40
	SA431-18		2018 Jun 19		1:20,480		1:80
4	SA345-18	Mid-May 2018	2018 May 21	Positive (35.44)	1:640	Positive	1:40
	SA387-18		2018 Jun 4		1:2,560		1:40
	SA435-18		2018 Jun 19		1:20,480		1:80
5	SA346-18	Mid-May 2018	2018 May 21	Negative	1:320	Positive	1:10
	SA384-18		2018 Jun 4		1:2,560		1:40
	SA433-18		2018 Jun 19		1:10,240		1:40
6	SA347-18	Mid-May 2018	2018 May 21	ND	Negative	Negative	Negative
	SA393-18		2018 Jun 4		Negative		Negative
7	SA385-18	No symptoms	2018 Jun 4	ND	1:2,560	Positive	1:80
	SA436-18		2018 Jun 4		1:10,240		1:80
8	SA388-18	No symptoms	2018 Jun 4	ND	1:1,280	Positive	1:20
	SA434-18		2018 Jun 19		1:10,240		1:40
9	SA389-18	Mid-May 2018	2018 Jun 4	ND	1:2,560	Positive	1:20
	SA430-18		2018 Jun 19		1:20,480		1:80
10	SA390-18	Mid-May 2018	2018 Jun 4	ND	1:2,560	Positive	Negative
	SA432-18		2018 Jun 19		1:20,480		1:40

*C_t, cycle threshold; HAI, hemagglutination inhibition; ND, not done; RVFV, Rift Valley fever virus; VNT, virus neutralization titer.
†Exact date of symptom onset could not be obtained, but all patients with symptoms estimated the date to be around the middle of May.
‡PCR was performed only on whole blood samples collected from 4 patients.

Appendix Table 2. RVFV sequence fragments obtained from sample SA344-18, South Africa, May 2018*

RVFV genome segment	Nucleotide position of fragments obtained
Large segment	63–292, 898–1455, 1457–2301, 2303–2327, 2653–3690, 3726–3795, 3797–4846, 4240–6398
Medium segment	27–207, 419–1756, 1929–3750
Small segment	18–793

RVFV, Rift Valley fever virus.

Appendix Table 3. Isolates used for phylogenetic analysis of Rift Valley fever virus isolate SA344-18, South Africa, May 2018

			GenBank accession no.		
Isolate name	Country of isolation	Year	Large segment	Medium segment	Small segment
73HB1230_CAR_1973	Central African Republic	1973	DQ375425	DQ380221	DQ380172
2269–74_ZIM_1974	Zimbabwe	1974	DQ375434	DQ380222	DQ380173
272659_MAU_2015	Mauritania	2015	KY366327	KY366326	KY366325
SA-75_RSA_1975	South Africa	1975	DQ375428	DQ380189	DQ380175
Beijing-01_PRC_2016	China	2016	KX611605	KX611606	KX611607
ZM-657_EGY_1978	Egypt	1978	DQ375409	DQ380204	DQ380146
ZH-501_EGY_1977	Egypt	1977	DQ375406	DQ380200	DQ380149
ZS-6365_EGY_1979	Egypt	1979	DQ375410	DQ380205	DQ380145
VRL2250–74_ZIM_1974	Zimbabwe	1974	DQ375413	DQ380209	DQ380143
2000–10911_SAU_2000	Saudi Arabia	2000	DQ375401	DQ380197	DQ380170
SA01–1322_SAU_2001	Saudi Arabia	2001	KX096941	KX096942	KX096943
Kenya-9800523_KEN_1998	Kenya	1998	DQ375400	DQ380196	DQ380169

Isolate name	Country of isolation	Year	GenBank accession no.		
			Large segment	Medium segment	Small segment
73HB1449_CAR_1974	Central African Republic	1974	DQ375416	DQ380218	DQ380162
Hv-B375_CAR_1985	Central African Republic	1985	DQ375422	DQ380218	DQ380161
SA35–74_RSA_1974	South Africa	1974	JF784386	JF784387	JF784388
OS-1_MAU_1987	Mauritania	1987	DQ375398	DQ380186	DQ380180
ARD-38388_BFA_1983	Burkina Faso	1983	DQ375399	DQ380187	DQ380181
Kenya-128b-15_KEN_2006	Kenya	2006	KX096938	KX096939	KX096940
ArB1986_CAR_1969	Central African Republic	1969	KJ782457	KJ782456	KJ782455
Lunyo_UGA_1955	Uganda	1955	KU167027	KU167026	KU167025
Sudan-85–2010_SUD_2010	Sudan	2010	JQ820485	JQ820488	JQ820476
Sudan-2V-2007_SUD_2007	Sudan	2007	JQ820483	JQ820490	JQ820472
2008–00099_MAY_2008	Mayotte	2008	HE687304	HE687303	HE687302
2007000234_KEN_2007	Kenya	2007	JF326186	JF326191	JF326198
200803162_MAD_2008	Madagascar	2008	JF311368	JF311377	JF311386
Tan-001–07_TAN_2007	Tanzania	2007	HM586959	HM586970	HM586981
763–70_ZIM_1970	Zimbabwe	1970	DQ375426	DQ380188	DQ380174
Kenya-56_KEN_1956	Kenya	1956	DQ375427	DQ380190	DQ380176
Entebbe_UGA_1944	Uganda	1944	DQ375429	DQ380191	DQ380156
Smithburn_UGA_1944	Uganda	1944	DQ375430	DQ380193	DQ380157
Kenya-57_KEN_1951	Kenya	1951	DQ375431	DQ380192	DQ380155
SA-51_RSA_1951	South Africa	1951	DQ375433	DQ380195	DQ380158
Kenya-83_KEN_1983	Kenya	1983	DQ375402	DQ380198	DQ380171
MgH824_MAD_1979	Madagascar	1979	DQ375414	DQ380210	DQ380144
1260–78_ZIM_1978	Zimbabwe	1978	DQ375418	DQ380214	DQ380164
ANK-3837_Guinea_1981	Guinea	1981	DQ375420	DQ380215	DQ380165
ANK-6087_Guinea_1984	Guinea	1984	DQ375421	DQ380216	DQ380166
Kakamas_RSA_2009	South Africa	2009	JQ068144	JQ068143	JQ068142
25010_24_MAU_2010	Mauritania	2010	Not available	KM210509	KM210508

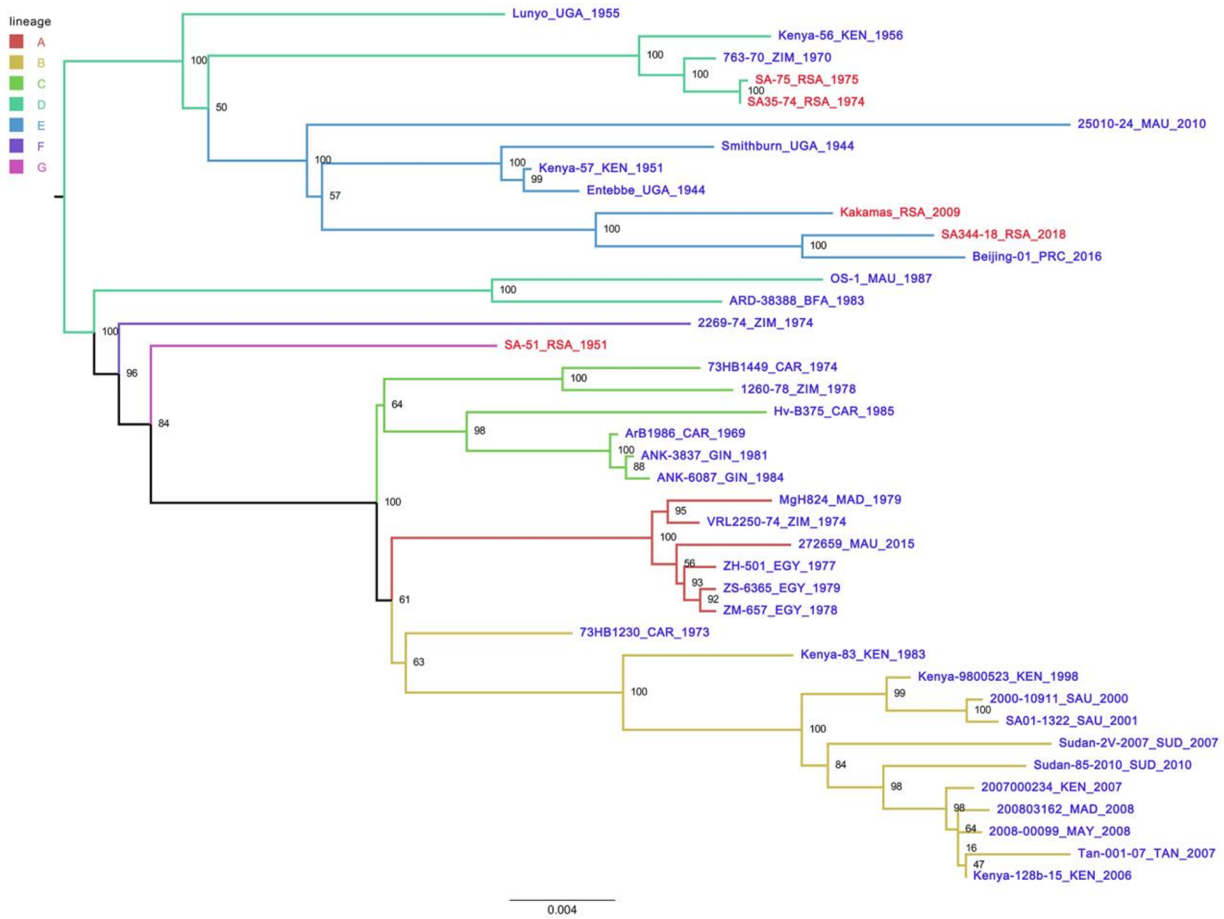

Appendix Figure 1. Maximum-likelihood tree showing the phylogeny of Rift Valley fever virus isolate SA344-18 based on the concatenated M segment, South Africa, May 2018. Sequence names from South Africa are colored in red font, and those from other countries are blue. Branches are colored according to lineage, based on the nomenclature of Bird et al. (*4*). Maximum-likelihood analysis was performed in RAxML; 10,000 bootstrap replicates were performed. Bootstrap values are shown at the nodes. Scale bar indicates nucleotide changes per base pair. BFA, Burkina Faso; CAR, Central African Republic; EGY, Egypt; GIN, Guinea; KEN, Kenya; MAD, Madagascar; MAU, Mauritania; MAY, Mayotte; PRC, China; RSA, South Africa; SAU, Saudi Arabia; SUD, Sudan; TAN, Tanzania; UGA, Uganda; ZIM, Zimbabwe.

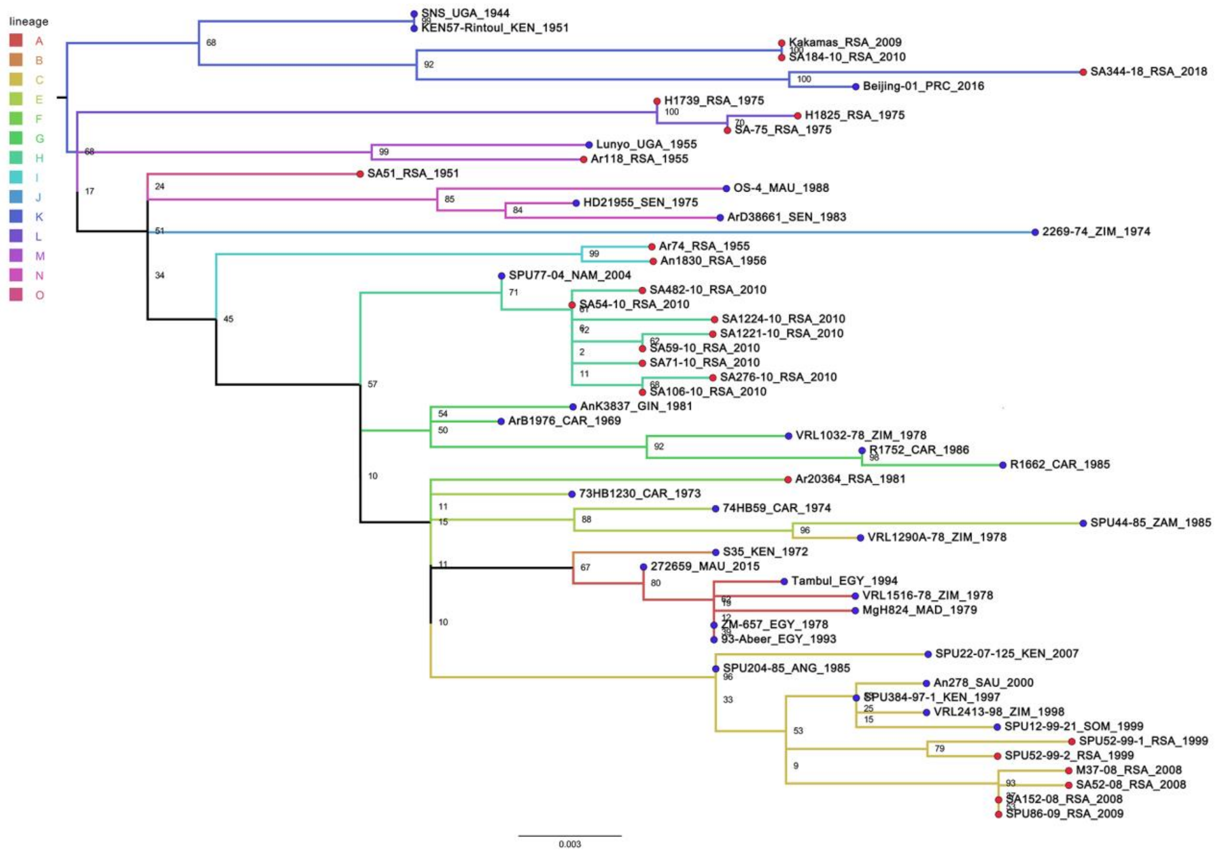

Appendix Figure 2. Maximum-likelihood tree showing the phylogeny of Rift Valley fever virus isolate SA344-18 based on the partial S segment, South Africa, May 2018. Sequence names from South Africa are colored in red font, while those from other countries are blue. Branches are colored according to lineage, based on the nomenclature of Bird et al. (*4*). Maximum-likelihood analysis was performed in RAxML; 10,000 bootstrap replicates were performed. Bootstrap values are provided at nodes. Scale bar indicates nucleotide changes per base pair. CAR, Central African Republic; EGY, Egypt; GIN, Guinea; KEN, Kenya; MAD, Madagascar; MAU, Mauritania; NAM, Namibia; PRC, China; RSA, South Africa; SAU, Saudi Arabia; SEN, Senegal; SOM, Somalia; UGA, Uganda; ZAM, Zambia; ZIM, Zimbabwe.
